# Effectiveness of edaravone in preventing contrast‐induced nephropathy in high‐risk patients undergoing coronary angiography: A randomized, double‐blind trial

**DOI:** 10.1002/prp2.1228

**Published:** 2024-07-02

**Authors:** Azam Esmailnejad, Sepideh Zununi Vahed, Seyyedeh Mina Hejazian, Naser Aslanabadi, Hassanali Lotfollahhi Gharakhanlu, Majid Saraei, Ahmad Ahmadzadehpournaky, Kasra Ardalan, Mohammadreza Ardalan, Samad Ghaffari Bavil

**Affiliations:** ^1^ Cardiovascular Research Center Tabriz University of Medical Sciences Tabriz Iran; ^2^ Kidney Research Center Tabriz University of Medical Sciences Tabriz Iran; ^3^ School of Pharmacy and Pharmaceutical Sciences Islamic Azad University Teheran Iran

**Keywords:** antioxidants, chronic kidney injury, contrast media, contrast‐induced‐acute kidney injury, edaravone

## Abstract

Contrast‐induced nephropathy (CIN) is a serious complication that occurs subsequent to the administration of contrast media for therapeutic angiographic interventions. As of present, no effective therapy exists to prevent its occurrence. This single‐center double‐blind randomized controlled trial aimed to evaluate the effect of edaravone, an antioxidant, in a group of high‐risk patients undergoing coronary angiography. Ninety eligible patients with chronic kidney disease Stages 3–4 were randomly assigned to either the control group (*n* = 45) or the intervention group (*n* = 45). In the intervention group, one dosage of edaravone (60 mg) in 1 L of normal saline was infused via a peripheral vein 1 h prior to femoral artery‐directed coronary angiography. Patients in the control group received an equal amount of infusion in their last hour before angiography. Both groups received intravenous hydration with 0.9% sodium 1 mL/kg/h starting 12 h before and continuing for 24 h after angiography. The primary outcome measure was the onset of CIN, defined as a 25% increase in serum creatinine levels 120 h after administration of contrast media. The occurrence of CIN was observed in 5.5% (*n* = 5) of the studied population: 2.2% of patients in the intervention group (*n* = 1) and 8.9% of controls (*n* = 4). However, this difference was not statistically significant. Administration of a single dosage of edaravone 1 h prior to infusion of contrast media led to a reduction in the incidence of CIN. Further investigations, employing larger sample sizes, are warranted to gain a comprehensive understanding of its efficacy.

## Graphical abstract

CONSORT diagram. The eligibility assessment of enrolled participants in the study.

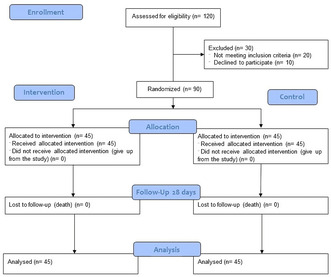

**Abbreviations**
BP: Blood pressure
BMI: Body mass index
BUN: Blood urea nitrogen
CIN: Contrast‐inducednephropathy
CKD: Chronic kidney disease
EF: Ejection fraction
eGFR: Estimated glomerular filtration rate
IHD: Ischemic heart disease
INR: International Normalized Ratio
LVEF: Left ventricular ejection fraction
NAC: N‐acetylcysteine
NSTEMI: non‐STEMI
SD: Standard deviation
STEMI: St elevation myocardial infarction
PCI: Percutaneous coronary intervention
ROS: Reactive oxygen species
UA: Unstable angina
WBC: White blood cell

## 1 INTRODUCTION

Contrast‐induced nephropathy (CIN) or acute kidney injury (CI‐AKI) is a common and potentially serious complication that can occur after the administration of contrast media for therapeutic angiographic intervention or intravascular diagnostic procedures.^1^ It is characterized by a sudden decline in kidney function following contrast exposure, leading to an increase in serum creatinine levels (≥1.5 times the baseline value or ≥0.3 mg/dL within 48 h) or a decrease in urine output (<0.5 mL/kg/h for over 6 consecutive hours).^1^

After the AKI induced by hypotension and cardiac surgery, CIN is the third cause of hospital‐acquired AKI.^2^ Depending on the demographic and risk factors, the incidence of CIN is ranged from 3% to 50%.^3^, ^4^ In the general healthy population, the incidence of CIN is low; however, chronic kidney disease (CKD) and diabetes patients are at high risk for CIN. Dehydration, advanced age, hypertension, hyperuricemia, heart failure, multiple myeloma, higher volume of contrast, and the use of nephrotoxic drugs are other risk factors for developing CIN.^5^ CIN can be recovered; however, some patients (~15%) may require dialysis temporarily^6^ and the CIN mortality rate ranges from 3.8% to 64%.^7^, ^8^, ^9^

CIN is a complex condition with multiple pathogenic mechanisms involved. Direct cellular toxicity of contrast media on renal tubular cells leads to cell death and impairment of their function. This can disrupt the delicate balance of electrolytes and fluid homeostasis in the kidneys, resulting in impaired filtration and excretion of waste products. Inflammation, vasoconstriction, and dysregulation of molecular pathways all contribute to the development of CIN.^10^, ^11^ Another important mechanism involved in CIN is the induction of oxidative stress. Contrast media can generate reactive oxygen species (ROS) within the kidney, overwhelming the endogenous antioxidant defense mechanisms. ROS, in turn, can damage the cellular structures, leading to cellular dysfunction and death.^10^, ^11^ Prevention and management strategies for CIN include adequate hydration before and after the procedure, minimizing the use of contrast agents, using lower doses of contrast agents when possible, and considering alternative imaging modalities in high‐risk individuals.^12^ Antioxidants, such as ascorbic acid and N‐acetylcysteine (NAC), have been investigated as potential protective agents against CIN due to their ability to reduce oxidative stress and inflammation. Therefore, it seems that the auxiliary use of drugs with antioxidant effects can prevent the damage of the contrast agent.

Edaravone is a free‐radical scavenger and an antioxidant that targets peroxyl radicals of ROS and has shown clinical efficacy in patients with acute ischemic stroke. Given the high risk of CIN in CKD patients and the need for the development of preventive therapeutic strategies to mitigate the risk of CIN, this clinical trial was conducted to evaluate the impact of edaravone in preventing CIN in patients with CKD (3–4 stages).

## 2 METHODS

### 2.1 Study population and protocol

The study was a single‐center, double‐blind, randomized placebo‐controlled trial of edaravone in CKD patients who were about to undergo coronary angiography. Participants were recruited from Shahid Madani Heart Hospital in Tabriz, Iran, between June 2021 and November 2022. Eligible candidates for the study included patients with CKD at Stages 3a, 3b, and 4 or with 59 ≤ eGFR (Estimated glomerular filtration rate) >15, and who were candidates for undergoing coronary angiography. Exclusion criteria encompassed pregnancy, lactation, age over 80 years, hypersensitivity to contrast agents, a history of asthma (as it may provoke asthma episodes in susceptible individuals), and severe liver damage (defined as a 5‐fold increase in liver enzymes). Since edaravone contains sodium bisulfite, which makes high potential for anaphylactic reactions, patients with asthma were excluded from this study.^13^

### 2.2 Randomization and intervention

**FIGURE 1:**
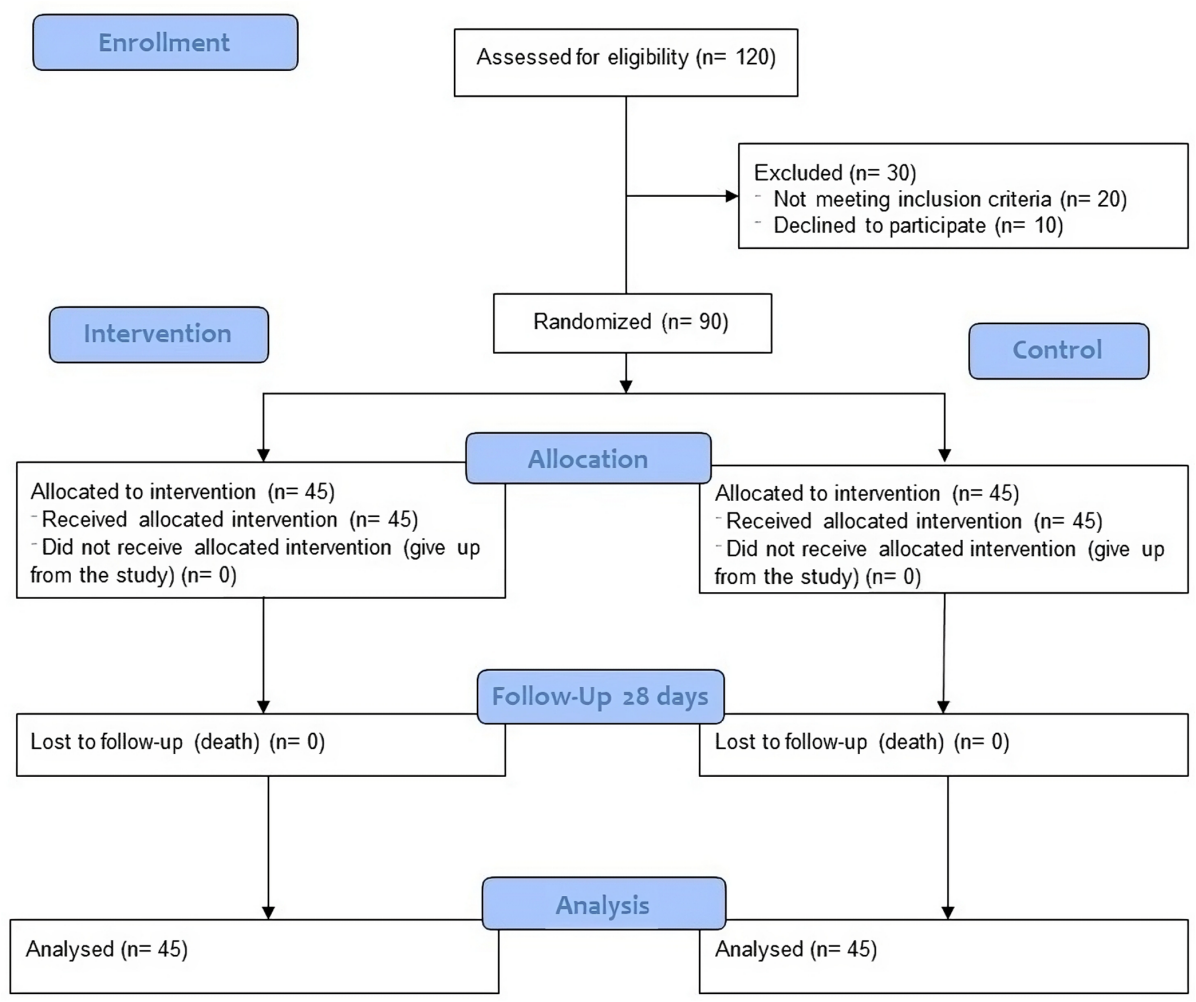
CONSORT diagram.

Participants who met the aforementioned criteria were randomly allocated to either the placebo or intervention groups, in a 1:1 ratio, utilizing computer‐generated random numbers (Figure 1). Throughout the analysis, both the statisticians and patients remained unaware of the assigned group. Ninety eligible patients were randomly assigned to the control group (*n* = 45) or the intervention group (*n* = 45). Due to the limited timeframe available for hydration during immediate angiography, the administration of a contrast agent may potentially result in nephropathy, particularly in patients with renal dysfunction. In order to ensure the reliability of our findings, our study excluded cases requiring urgent percutaneous coronary intervention (PCI) for ST‐segment elevation myocardial infarction (STEMI) that did not allow for sufficient time for appropriate hydration and drug administration. Hydration was administered to all patients who had sufficient time for preparation prior to angiography. They were given a 0.9% saline solution at a rate of 1 mL/kg/h for 12 h before and 24 h after the angiography procedure. In the intervention group, a single dose of edaravone (60 mg, Zistdaru Co. Tehran, Iran) was intravenously infused within 60 min, 1 h prior to the angiography.

Patients in the control group received a placebo under similar conditions. Coronary angiography was performed by an expert utilizing a standard technique through the femoral artery for all patients. To minimize variables affecting CIN, a non‐ionic, low‐osmolarity iodinated contrast agent (Omnipaqe300) was employed during the procedure. The quantity of contrast material used for each patient was documented and compared between the two groups. No alterations were made to the medication regimen, unless necessary. Furthermore, the patients in both groups were categorized into four groups: STEMI, non‐ST‐elevation myocardial infarction (NSTEMI), unstable angina (UA), and elective cases.

In the case of all patients, prior to the administration of angiography, the echocardiography procedure was performed with precision, thereby facilitating the accurate measurement of the ejection fraction (EF) value through the employment of the TTE modality. Since the patients entered the study after a definite acute coronary syndrome status, the EF value exhibited no alterations during the hospitalization and none of the patients' condition worsened during the hospitalization. For instance, patients suffering from unstable angina did not transit into STEMI, thereby ensuring the EF value remained constant throughout the entirety of hospitalization and did not undergo any dynamic modifications.

### 2.3 Biochemical parameters

The demographic information of the patients including age, gender, height, weight, BMI (body mass index), platelet, sodium, WBC (white blood cell), LVEF (left ventricular ejection fraction), INR (International Normalized Ratio), hemoglobin, and smoking history was recorded. The underlying diseases including diabetes, high blood pressure, hyperlipidemia, ischemic heart disease, and heart failure were recorded. The patients were followed up precisely for 5 days.

### 2.4 Study endpoints and definitions

The primary outcome of the study was the incidence of CIN, defined as a relative 25% increase in serum creatinine from baseline within 48 h after contrast media administration.^14^

### 2.5 Statistical analysis

The statistical analysis was performed using IBM SPSS statistics software (version 22; Armonk). Data for continuous variables data were presented as mean ± standard deviation (SD), and categorical variables were expressed as frequency (%). To assess the differences between these two groups, Independent sample *t*‐tests, paired sample *t*‐test, or Mann–Whitney *U* test were applied. A *p*‐value <.05 was considered statistically significant.

## 3 RESULTS AND DISCUSSION

### 3.1 Subjects

In the present clinical trial, a total of 90 CKD patients undergoing coronary angiography were enrolled and all completed the study. Patients were randomly (1:1) divided into two intervention and control groups. None of our patients demonstrated indications for immediate intervention, thereby providing a suitable opportunity for hydration and drug administration. The average age of the patients was 65.17 ± 9.981 years. Most of the included patients were male (*n* = 66). There were insignificant differences between the two groups for baseline characteristics including age, BMI, hemoglobin, platelets, potassium, sodium levels, and INR (*p* > .05). However, the percentage of LVEF [50 (25–60) vs. 40 (20–55), *p* = .001] and amount of WBC [8 (4.40–20) vs. 12.80 (3.90–18.70) 10^9^ cells/L, *p* < .001] were statistically different in the intervention group compared to controls, respectively (Table 1). At the beginning of the study and before the intervention, it was found that there was no statistically significant difference in the eGFR between the control and intervention groups, and none of the patients in the two groups had an eGFR above 59 (*p* = .229). The amount of contrast agents used in patients who did not undergo PCI was statistically significant between the two groups (*p* = .008).

**TABLE 1:** Demographic and baseline clinical information of the participants.

Variables	Subgroups	All participants (*n* = 90)	Groups		*p*‐value
			Control (*n* = 45)	Intervention (*n* = 45)	
Gender	Male	66 (73.3)	38 (57.6)	28 (42.4)	**.031**
	Female	24 (26.7)	7 (29.2)	17 (70.8)	
Age (years)		65.17 ± 9.981	65.42 ± 10.94	64.9 ± 19.03	.810
Height (cm)		169 (151–185)	169 (151–178)	171 (155–185)	.218
Weight (kg)		78 (58–98)	78 (59–98)	78 (58–98)	.903
BMI (kg/m^2^)		26.4 (20.4–38.6)	26 (20.4–38.6)	26 (21–38)	.315
Systolic BP (mmHg)		134.6 ± 10.7	131.6 ± 13.5	135.1 ± 7.2	.807
Diastolic BP (mmHg)		77.9 ± 8.4	77.9 ± 8.4	74.8 ± 7.5	.650
eGFR (ml/min/1.73m^2^)		39.43 (15.45–55.22)	40.46 (21.86–55.22)	38.64 (10.45–49.88)	.229
Severity of heart disease	Elective	9 (10)	0	9 (100)	**<.001**
	UA	23 (25.6)	8 (34.8)	15 (65.2)	
	NSTEMI	23 (25.6)	12 (52.2)	11 (47.8)	
	STEMI	35 (38.9)	25 (71.4)	10 (28.6)	
PCI
	42 (46.7)	22 (52.4)	20 (47.6)	.833
Contrast (mg/mL)	PCI positive	120 (25–150)	120 (90–150)	110 (25–145)	.40
	PCI negative	30 (20–150)	30 (25–150)	27.50 (20–120)	**.008**
LVEF (%)		45 (20–60)	40 (20–55)	50 (25–60)	**.001**
WBC (10^9^/L)		9.85 (3.90–20)	12.80 (3.90–18.70)	8 (4.40–20)	**<.001**
Hb (mg/dL)		13.75 (8.20–18.80)	13.50 (10.40–18.00)	13.80 (8.20–18.80)	.728
Platelet (10^9^/L)		201.50 (104–592)	211 (128–437)	192 (104–592)	.345
Potassium (mg/dL)		4.26 ± 0.41	4.25 ± 0.45	4.28 ± 0.38	.756
Sodium (mg/dL)		139.87 ± 3.904	139.47 ± 2.95	140.27 ± 2.83	.193
INR		1.06 (0.9–1.45)	1.05 (0.90–1.32)	1.10 (0.96–1.45)	.563
Smoker		30 (33.3)	17 (43.3)	13 (56.7)	.503

*Note*: Data are presented as mean ± standard deviation or median (minimum–maximum). *t*‐test or Mann–Whitney tests were used to compare the results between the two groups. The categorical data were compared by chi‐square test and reported as number (percentage). *p*‐value < 0.05 was considered statistically significant.

Abbreviations: BP, blood pressure; BMI, body mass index; eGFR, estimated glomerular filtration rate; INR, International Normalized Ratio; LVEF, Left ventricular ejection fraction; STEMI, St elevation myocardial infarction; NSTEMI, non‐STEMI; PCI, percutaneous coronary intervention; UA, unstable angina; WBC, white blood cell.

Regarding the severity of the disease, 38.9% had STEMI, 25.6% had unstable angina, 25.6% had NSTEMI, and 10% of patients had an elective. The distribution of patients with different disease severity was statistically different between the two groups (*p* < .001). It was also found that 46.7% of patients underwent PCI, and the distribution of these people between the two groups was not statistically significant (*p* = .833), Table 1.

In subgroup analysis, the studied variables were compared within the control and intervention groups, separately. In the control subgroup analysis, the percentage of LVEF among patients with unstable angina was significantly higher than in other groups (*p* = .021). The same results were obtained in the intervention subgroup analysis (*p* = .021). In terms of other demographic and clinical parameters, there were no significant differences in our subgroup analysis (*p* ≥ .078), Table S1.

The underlying diseases of CKD patients undergoing angiography were also investigated. Most of the patients have hypertension (75.55%). A number of patients also had diabetes (44.44%), hyperlipidemia (41.1%), ischemic heart disease (27.77%), and heart failure (5.55%). No statistical difference was observed in terms of the underlying diseases between the two studied groups (*p* ≥ .392), Table 2. Subgroup analysis regarding the severity of the disease showed that most of the patients with high blood pressure (*p* = .039) and diabetes (*p* = .031) had STEMI (Table 2).

**TABLE 2:** The history of underlying diseases of the patients.

Groups	Underlying diseases				
	Hypertension	Diabetes	Hyperlipidemia	IHD	Heart failure
All participants	68 (75.55)	40 (44.44)	37 (4.11)	25 (27.77)	5 (5.55)
Edaravone	33 (48.5)	18 (45)	16 (43.2)	9 (36)	2 (40)
Control	35 (51.5)	22 (55)	21 (56.8)	16 (64)	3 (60)
*p*‐value	.807	.525	.392	.517	1
Elective	4 (5.9)	0	1 (2.7)	1 (4)	0
UA	18 (26.5)	10 (25)	8 (21.6)	4 (16)	2 (40)
NSTEMI	21 (30.9)	13 (32.5)	12 (32.4)	5 (20)	1 (20)
STEMI	25 (36.8)	17 (42.5)	16 (43.2)	15 (60)	2 (40)
*p*‐value	**.039**	**.031**	.165	.076	.936
CIN	4 (5.9)	3 (7.5)	1 (2.7)	1 (4)	0
Non‐CIN	64 (94.1)	37 (92.5)	36 (97.3)	24 (96)	5 (100)
*p*‐value	1	.652	.401	1	1

*Note*: The results were reported as number (percentage) and analyzed by chi‐square test. *p*‐value < 0.05 was considered statistically significant.

Abbreviations: CIN, contrast‐induced nephropathy; IHD, ischemic heart disease; NSTEMI, non‐STEMI; STEMI, St elevation myocardial infarction; UA, unstable angina.

Among the studied population, five patients (5.5%) developed CIN 5 days after the intervention, all of whom were men and had hypertension and diabetes compared to the non‐CIN group, *p* > .05 (Table 3). Age, BMI, hemoglobin level, platelet, and INR ratio were also insignificantly higher in patients with CIN compared to the patients who did not develop CIN (*p* > .05). The median amount of contrast agent used in the CIN group was less than that in the non‐ CIN group [35 (30–150) vs. 45 (20–150)] that was not statistically significant (*p* = .901) since undergoing PCI was high in the control group. However, no significant differences were observed regarding the amount of contract media between patients in the control and intervention groups who underwent PCI [120 (90–150) vs. 110 (25–145), *p* = 0.40], Table 3. Among five CIN patients, three patients had STEMI and two patients had NSTEMI.

**TABLE 3:** Comparing the studied variables between the groups with or without CIN.

Variables	Subgroups	Groups		*p*‐value
		Non‐CIN (*n* = 85)	CIN (*n* = 5)	
Gender	Male	61 (71.76)	5 (100)	.319
	Female	24 (28.24)	0	
Age (years)		65.01 ± 10.10	67.80 ± 7.98	.407
Height (cm)		169 (151–185)	169 (166–177)	.647
Weight (kg)		76 (58–98)	79 (78–91)	.086
BMI (kg/m^2^)		26 (20.4–37.6)	30 (26.4–35)	.075
PCI		41 (48.24)	1 (20)	.367
Groups	Control	41 (48.24)	4 (80)	.361
	Edaravone	44 (51.76)	1 (20)	
Severity of heart disease	Elective	9 (10.58)	0	.442
	UA	23 (27.06)	0	
	NSTEMI	21 (24.71)	2 (20)	
	STEMI	32 (37.65)	3 (60)	
Contrast (mL)		45 (20–150)	30 (30–150)	.901
LVEF (%)		45 (20–60)	40 (20–50)	.144
WBC (10^9^/L)		10 (3.9–20)	9.3 (405–16.5)	.647
Hb (mg/dL)		13.7 (8.2–18.8)	13.8 (10.7–16)	.833
Platelet (10^9^/L)		200 (104–592)	272 (163–306)	.195
Potassium (mg/dL)		4.25 ± 0.38	4.380.82 ± 0.82	**0.008**
Sodium (mg/dL)		139.89 ± 2.96	139.4 ± 1.51	.159
INR		1.06 (0.9–1.45)	1.11 (1–1.19)	.581
Smoker		28 (32.94)	2 (40)	1

*Note*: Data are presented as mean ± standard deviation or median (minimum–maximum). *t*‐test or Mann–Whitney tests were used to compare the results between the two groups. The categorical data were compared by chi‐square test and reported as number (percentage). *p*‐value < 0.05 was considered statistically significant.

Abbreviations: BMI, body mass index; CIN, contrast‐induced nephropathy; Hb, hemoglobin; INR, International Normalized Ratio; LVEF, Left ventricular ejection fraction; NSTEMI, non‐STEMI; PCI, percutaneous coronary intervention; STEMI, St elevation myocardial infarction; WBC, white blood cell; UA, unstable angina.

### 3.2 The effect of edaravone drug on renal factors

Four out of five CIN patients were from the control group. In examining the effect of the intervention on the kidney parameters in patients undergoing CIN, it was found that edaravone was able to protect the kidney against CIN, the serum level of creatinine in the intervention group was lower compared to the control group (*p* = .043). However, there was no statistically significant difference in the BUN level between the two groups (*p* = .926). It should be noted that at the beginning of the study, BUN (*p* = .183) and serum creatinine levels (*p* = .536) were not statistically significant between the two groups. It was also found that 5 days after the intervention, there was a statistically significant difference between the serum creatinine (*p* < .001) and BUN (*p* = .004) levels in the intervention group compared to the controls (*p* = .709 and *p* = .861, respectively), Table 4.

**TABLE 4:** The effect of edaravone on kidney function in the studied groups.

Kidney parameters	Intervention stage	Groups		*p*‐value
		Control group	Intervention group	
Creatinine	Before treatment	1.70 (1.45–2.80)	1.67 (1.4–4.2)	.536
	After treatment	1.66 (1.25–3.30)	1.50 (1.1–4.7)	**.043**
	*p*‐value	.709	**<.001**	‐
BUN	Before treatment	28 (13–27)	32 (10–84)	.183
	After treatment	27 (15–74)	25 (14–97)	.926
	*p*‐value	.861	**.004**	‐

*Note*: Data presented as median (minimum‐maximum). Mann–Whitney tests were used to compare the results between the groups. *p*‐value < 0.05 was considered statistically significant.

Abbreviation: BUN, blood urea nitrogen.

## 4 DISCUSSION

The primary outcome of this investigation revealed that edaravone might potentially contribute to the reduction in the occurrence of CIN in individuals at high risk (CKD Stages 3 and 4) who undergo coronary angiography, although this finding did not reach statistical significance.

Individuals suffering from CKD face an increased vulnerability to developing cardiovascular ailments, necessitating the implementation of coronary interventions when compared to the general population.^15^ Several studies have highlighted the increased risk of CIN developing in the CKD population compared to those without CKD.^16^ To date, no effective preventive strategies for CIN have been established; thus, the key lies in implementing effective protective strategies to minimize its occurrence in high‐risk patients.

A significant number of Meta‐analysis studies have delineated the potential advantages of antioxidants, pharmacological interventions (Statin), and vitamins (C, E, D) in addition to hydration in the reduction of the risk associated with CIN.^17^, ^18^, ^19^, ^20^ Nevertheless, the PRESERVE trial, which encompasses a substantial sample size, has failed to identify any nephroprotective effect of N‐acetylcysteine (NAC) against CIN.^21^ There exists a limited body of evidence regarding the utilization of antioxidants for the prevention of CIN in patients with CKD.^22^, ^23^ A salutary influence of NAC in averting CIN has been documented,^24^ particularly in those with more severe CKD.^23^ Administration of vitamin E (α‐ or γ tocopherol) in combination with saline (0.9%) infusion has also been indicated to protect against CIN in CKD patients undergoing coronary procedures.^25^, ^26^ Moreover, prophylactic vitamin E (high‐dose 600 mg 12 h before + 400 mg 2 h before coronary angiography) combined with saline infusion could significantly decrease the incidence of CIN compared to placebo in CKD patients.^26^ It is crucial to note, however, that despite these findings, there remains a debate and inconsistency regarding the efficacy of antioxidants in patients with CKD Stages 3 and 4.

Based on the existing findings, edaravone, as a free‐radical scavenger, reduces oxidative stress and prevents cellular damage caused by ROS. Moreover, edaravone exhibits the ability to safeguard organs against inflammation, radiation, and ischemia–reperfusion injury.^27^, ^28^, ^29^, ^30^ In animal models, pre‐treatment with edaravone has been shown to attenuate renal injury and histologic changes after contrast administration via increasing renal antioxidant capacity.^31^ Correspondingly, in the present study, the administration of edaravone 1 h before contrast usage resulted in a decrease in the incidence of CIN, with only one patient experiencing CIN development compared to four patients in the control group. Interestingly, a comparison of the median serum creatinine levels between the edaravone and control groups revealed a significant difference, with lower serum creatinine and BUN levels observed in the former group. These findings further support the potential efficacy of edaravone in mitigating CIN. The beneficial effects of edaravone may stem from its antioxidative, anti‐inflammatory properties, and preservation of renal microcirculation, thereby mitigating the occurrence of CIN. However, it is important to note that the evidence remains limited, as our study represents the first of its kind in this field.

From a clinical perspective, the results of this study are promising; however, certain limitations must be acknowledged. The main constraint of this trial was the small sample size. Subsequent research is necessary to validate these preliminary findings and to establish the optimal dosage, timing, and duration of edaravone treatment for preventing CIN. Moreover, con‐founding factors including effective medications in preventing CIN like statins, bicarbonate, and NAC should be considered in the beginning of the study and compared between groups. Well‐designed randomized controlled trials conducted across multiple centers and involving a large sample size are imperative for drawing definitive conclusions regarding the beneficial effects of edaravone in the CKD population.

## 5 CONCLUSION

Edaravone has the potential to reduce the incidence of CIN in individuals with CKD who undergo angiography. However, it is worth noting that this reduction did not reach statistical significance. Edaravone may exert a preventive effect against CIN through the reduction of oxidative stress and protection against cellular damage. Nonetheless, further research with a large sample size and rigorous design is inssdispensable for a comprehensive understanding of the effectiveness and optimal implementation of edaravone in this context.

## AUTHOR CONTRIBUTIONS

MA and SGB: Design the work. AE, MS, NA, HLG, and AA: Patients' allocation and treatment, and data collection. MH and KA: Data analysis. MA and SZV: Data interpretation, drafting, and revising the work.

## FUNDING INFORMATION

This study was financially supported by Tabriz University of Medical Sciences (#Grant_No: 65632).

## CONFLICT OF INTEREST STATEMENT

The authors report no relationships that could be construed as a conflict of interest.

## ETHICS STATEMENT

The Ethics Review Board of Tabriz University of Medical Sciences approved the study protocol (IR.TBZMED.REC.1399.1183). This study was conducted according to the standards of the Declaration of Helsinki and current ethical guidelines.

## CONSENT TO PARTICIPATE

Written consent was obtained from all participants.

## CLINICAL TRIAL REGISTRATION

The study was registered at the Iranian Registry of Clinical Trials website (http://www.irct.ir, registration number: IRCT20201108049311N5).

## Supporting information


Table S1.

## Data Availability

All the data generated or analyzed during this study are available.
