# Supplementary material for: Effectiveness of edaravone in preventing contrast‐induced nephropathy in high‐risk patients undergoing coronary angiography: A randomized, double‐blind trial
Source: Pharmacol Res Perspect. 2024 Jul 2;12(4):e1228. doi: 10.1002/prp2.1228 (PMC11219510; doi:10.1002/prp2.1228)
Supplement: Supplementary file 1 — Table S1. [file PRP2-12-e1228-s001.docx]

**Supplementary Table 1. Clinical findings in the studied groups with different heart disease severity**

| **P-value** | **Severity of the heart disease** | | | | **Subgroups** | | **Variables** |
| --- | --- | --- | --- | --- | --- | --- | --- |
|  | **STEMI** | **NSTEMI** | **UA** | **Elective** |  |  |  |
| **<0.001** | 25 (71.43) | 12 (52.17) | 8 (34.78) | 0 | **Control** | | **Number of patients** |
|  | 10 (28.57) | 11 (47.83) | 15 (65.21) | 9 (100) | **Intervention** | |  |
| 0.140 | 23 (92) | 8 (66.67) | 7 (87.5) | - | **Male** | **Control** | **Gender** |
|  | 2 (8) | 4 (33.33) | 1 (12.5) | - | **Female** |  |  |
| 0.845 | 7 (70) | 7 (63.64) | 8 (53.33) | 6 (21.4) | **Male** | **Intervention** |  |
|  | 3 (30) | 4 (36.36) | 7 (46.67) | 3 (17.6) | **Female** |  |  |
| 0.457 | 63.60±10.54 | 67.25±12.59 | 68.37±9.70 | - | **Control** | | **Age (Years)** |
| 0.193 | 69.80±5.41 | 64±9.87 | 64.67±9.52 | 61±9.32 | **Intervention** | |  |
| 0.078 | 170 (161-178) | 167.5 (154-175) | 166.5 (151-176) | - | **Control** | | **Height (cm)** |
| 0.627 | 177 (155-185) | 172 (157-184) | 168 (156-183) | 168 (156-183) | **Intervention** | |  |
| 0.390 | 76 (59-98) | 80 (67-94) | 77 (65-88) | - | **Control** | | **Weight (Kg)** |
| 0.502 | 80 (65-91) | 76 (68-82) | 75 (58-98) | 71 (58-98) | **Intervention** | |  |
| 0.148 | 26.4 (20.4-32.7) | 27.45 (24-36.7) | 26.5 (23.6-38.6) | - | **Control** | | **BMI (Kg/m^2^)** |
| 0.677 | 28 (21-35) | 25 (23-33) | 27 (21-38) | 24 (21-36) | **Intervention** | |  |
| 0.341 | 14 (56) | 6 (50) | 2 (25) | - | **Control** | | **PCI** |
| 0.415 | 5 (50) | 7 (63.64) | 5 (33.33) | 3 (33.33) | **Intervention** | |  |
| 0.840 | 120 (90-145) | 115 (100-150) | 125 (120-130) | - | **PCI positive** | **Control** | **Contrast (mg/ml)** |
| 0.552 | 30 (25-50) | 30 (25-30) | 35 (25-150) | - | **PCI negative** |  |  |
| 0.732 | 112.50 (25-130) | 110 (100-125) | 110 (90-135) | 140 (90-145) | **PCI positive** | **Intervention** |  |
| 0.315 | 30 (25-120) | 27.5 (20-30) | 27.50 (20.35) | 20 (20-30) | **PCI negative** |  |  |
| **0.021** | 35 (20-55) | 45 (30-55) | 50 (20-55) | - | **Control** | | **LVEF (%)** |
| **<0.001** | 37.5 (25-45) | 45 (30-55) | 55 (30-60) | 50 (40-55) | **Intervention** | |  |
| 0.930 | 12.6 (4.5-18.3) | 12.9 (6-18.7) | 11.95 (3.9-17) | - | **Control** | | **WBC (10^9^/L)** |
| **0.031** | 9.65 (5.9-16) | 9.9 (4.7-16) | 7.5 (5.6-20) | 7.3 (4.4-13.3) | **Intervention** | |  |
| 0.432 | 13.9 (10.50-17.10) | 13.75 (10.40-18) | 13.4 (11.70-14.10) | - | **Control** | | **Hb (mg/dl)** |
| 0.255 | 13.15 (11.2-18.8) | 12.5 (8.2-16.4) | 13.9 (10.3-15) | 14.5 (11.8-18.7) | **Intervention** | |  |
| 0.630 | 209 (128-316) | 199 (138-437) | 234 (168-309) | - | **Control** | | **Platelet (10^9^/L)** |
| 0.803 | 211.5 (146-345) | 182 (104-592) | 191 (110-297) | 192 (110-305) | **Intervention** | |  |
| 0.175 | 4.20±0.48 | 4.45±0.46 | 4.10±0.25 | - | **Control** | | **Potassium(mg/dL)** |
| 0.943 | 4.33±0.34 | 4.33±0.52 | 4.25±0.34 | 4.3±0.31 | **Intervention** | |  |
| 0.748 | 139.68±2.99 | 139.5±3.11 | 138.75±2.81 | - | **Control** | | **Sodium (mg/dL)** |
| 0.267 | 141.1±2.33 | 141.18±2.89 | 139.33±3.3 | 139.8±2.1 | **Intervention** | |  |
| 0.551 | 1.05 (0.9-1.32) | 1 (0.9-1.2) | 1.08 (1-1.28) | - | **Control** | | **INR** |
| 0.090 | 1.13 (1-1.35) | 1.12 (1-1.15) | 1 (0.9-1.2) | 1.1 (0.96-1.45) | **Intervention** | |  |
| 0.401 | 7 (28) | 6 (50) | 4 (50) | - | **Control** | | **Smoker** |
| 0.148 | 2 (20) | 4 (36.36) | 2 (13.33) | 5 (38.5) | **Intervention** | |  |

Data are presented as mean ± standard deviation or median (minimum-maximum). T-test or Mann-Whitney tests were used to compare the results between the two groups. The categorical data were compared by chi-square test and reported as number (percentage). BMI: Body mass index, Hb: hemoglobin, INR: International Normalized Ratio, LVEF: Left ventricular ejection fraction, STEMI: St elevation myocardial infarction, NSTEMI: non-STEMI, PCI: Percutaneous coronary intervention, WBC: white blood cell, UA: unstable angina.
